# Genomic Characterization of Carbapenem-Resistant Acinetobacter baumannii (CRAB) in Mechanically Ventilated COVID-19 Patients and Impact of Infection Control Measures on Reducing CRAB Circulation during the Second Wave of the SARS-CoV-2 Pandemic in Milan, Italy

**DOI:** 10.1128/spectrum.00209-23

**Published:** 2023-03-28

**Authors:** Davide Mangioni, Valeria Fox, Liliane Chatenoud, Matteo Bolis, Nicola Bottino, Lisa Cariani, Flaminia Gentiloni Silverj, Caterina Matinato, Gianpaola Monti, Antonio Muscatello, Antonio Teri, Leonardo Terranova, Alessandra Piatti, Andrea Gori, Giacomo Grasselli, Nino Stocchetti, Claudia Alteri, Alessandra Bandera

**Affiliations:** a Infectious Diseases Unit, Foundation IRCCS Ca’ Granda Ospedale Maggiore Policlinico di Milano, Milan, Italy; b Department of Pathophysiology and Transplantation, University of Milano, Milan, Italy; c Department of Oncology and Hemato-Oncology, University of Milan, Milan, Italy; d Istituto di Ricerche Farmacologiche Mario Negri IRCCS, Milan, Italy; e Department of Anaesthesia, Critical Care and Emergency, Foundation IRCCS Ca’ Granda Ospedale Maggiore Policlinico di Milano, Milan, Italy; f Microbiology Laboratory, Clinical Laboratory, Foundation IRCCS Ca’ Granda Ospedale Maggiore Policlinico di Milano, Milan, Italy; g Medical Direction, Foundation IRCCS Ca’ Granda Ospedale Maggiore Policlinico di Milano, Milan, Italy; h Department of Anesthesia and Intensive Care, ASST Grande Ospedale Metropolitano Niguarda, Milan, Italy; i Department of Internal Medicine, Respiratory Unit and Adult Cystic Fibrosis Center, Foundation IRCCS Cà Granda Ospedale Maggiore Policlinico, Milan, Italy; j Department of Anaesthesia and Critical Care, Neuroscience Intensive Care Unit, Foundation IRCCS Ca’ Granda Ospedale Maggiore Policlinico di Milano, Milan, Italy; Taichung Veterans General Hospital

**Keywords:** *Acinetobacter baumannii* CRAB, genomic surveillance, infection prevention and control (IPC), intensive care unit (ICU), phylogenetic analysis, whole-genome sequencing (WGS)

## Abstract

COVID-19 has significantly affected hospital infection prevention and control (IPC) practices, especially in intensive care units (ICUs). This frequently caused dissemination of multidrug-resistant organisms (MDROs), including carbapenem-resistant Acinetobacter baumannii (CRAB). Here, we report the management of a CRAB outbreak in a large ICU COVID-19 hub Hospital in Italy, together with retrospective genotypic analysis by whole-genome sequencing (WGS). Bacterial strains obtained from severe COVID-19 mechanically ventilated patients diagnosed with CRAB infection or colonization between October 2020 and May 2021 were analyzed by WGS to assess antimicrobial resistance and virulence genes, along with mobile genetic elements. Phylogenetic analysis in combination with epidemiological data was used to identify putative transmission chains. CRAB infections and colonization were diagnosed in 14/40 (35%) and 26/40 (65%) cases, respectively, with isolation within 48 h from admission in 7 cases (17.5%). All CRAB strains belonged to Pasteur sequence type 2 (ST2) and 5 different Oxford STs and presented *bla*_OXA-23_ gene-carrying Tn*2006* transposons. Phylogenetic analysis revealed the existence of four transmission chains inside and among ICUs, circulating mainly between November and January 2021. A tailored IPC strategy was composed of a 5-point bundle, including ICU modules’ temporary conversion to CRAB-ICUs and dynamic reopening, with limited impact on ICU admission rate. After its implementation, no CRAB transmission chains were detected. Our study underlies the potentiality of integrating classical epidemiological studies with genomic investigation to identify transmission routes during outbreaks, which could represent a valuable tool to ensure IPC strategies and prevent the spread of MDROs.

**IMPORTANCE** Infection prevention and control (IPC) practices are of paramount importance for preventing the spread of multidrug-resistant organisms (MDROs) in hospitals, especially in the intensive care unit (ICU). Whole-genome sequencing (WGS) is seen as a promising tool for IPC, but its employment is currently still limited. COVID-19 pandemics have posed dramatic challenges in IPC practices, causing worldwide several outbreaks of MDROs, including carbapenem-resistant Acinetobacter baumannii (CRAB). We present the management of a CRAB outbreak in a large ICU COVID-19 hub hospital in Italy using a tailored IPC strategy that allowed us to contain CRAB transmission while preventing ICU closure during a critical pandemic period. The analysis of clinical and epidemiological data coupled with retrospective genotypic analysis by WGS identified different putative transmission chains and confirmed the effectiveness of the IPC strategy implemented. This could be a promising approach for future IPC strategies.

## INTRODUCTION

Acinetobacter baumannii is one of the leading pathogens in intensive care units (ICUs) worldwide (1). It can either be detected as colonization on skin, mucosa, and biological fluids or cause severe infections, especially in fragile patients with indwelling devices or on mechanical ventilation (2, 3).

The ability of A. baumannii to spread through person-to-person transmission and to survive for prolonged periods on surfaces poses a risk for nosocomial outbreaks (4). Moreover, the majority of clinically relevant strains of A. baumannii are characterized by multiple-antibiotic resistance, including resistance to carbapenems, the last-line antimicrobials for Gram-negative bacteria. According to the Centers for Disease Control and Prevention, carbapenem-resistant A. baumannii (CRAB) is listed as an “urgent threat” because of its large diffusion, the lack of effective treatment options, and high mortality rates (5–8).

The coronavirus disease 2019 (COVID-19) pandemic at its peak caused dramatic challenges in the infection prevention and control (IPC) practices and antimicrobial stewardship programs (ASPs) in all health care settings (9). This had a strong impact on the dissemination of multidrug-resistant organisms (MDROs) within hospitals, especially in ICUs (10, 11). Several outbreaks of MDROs in COVID units have been described in the literature, including outbreaks of CRAB (12–15). Yet, in high-prevalence areas such as Southern and Eastern Europe, where prevalence of carbapenem resistance in A. baumannii is equal to or above 50% (16), difficulties exist in distinguishing nosocomial outbreaks from circulation between hospitals of CRAB based only on clinical and phenotypic characteristics. This difference is crucial for applying appropriate IPC measures depending on the scenario, especially in the ICU setting (i.e., unit closure, environmental culturing and cleaning, and cohorting of CRAB patients in case of nosocomial outbreak, as well as preventive isolation and universal screening at admission if circulation between hospitals occurs) (17, 18).

Here, we share our experience in the prevention and management of a CRAB outbreak faced during COVID-19 pandemic in a large ICU COVID-19 hub hospital in Italy. We later performed a genotypic analysis of CRAB isolates and defined transmission chains by integrating genome sequencing with epidemiological variables.

## RESULTS

### Study population.

Between October 2020 and May 2021, 40 patients had positive cultures for CRAB (see Fig. S1 in the supplemental material). The demographic and clinical features of the study population are detailed in Table 1. The median age was 67.5 years (interquartile range [IQR; Q1 to Q3], 62 to 72 years), 4/40 (10%) were female, half of the study population was obese (body mass index [BMI] of >30 kg/m^2^), and 32/40 (80%) had at least one comorbidity.

**TABLE 1:** Demographic and clinical features of the study population composed by 40 patients with carbapenem-resistant Acinetobacter baumannii infection or colonization

Demographic or clinical parameter^*a*^	Result for parameter shown^*b*^
Demographics	
Age, yr (IQR)	67.5 (62–72)
Female, no. (%)	4 (10.0)
BMI, score (IQR)	30 (27–32)
Comorbidity, no. (%)	
Hypertension	25 (62.5)
Cardiovascular disease	4 (10.0)
Diabetes mellitus	7 (17.5)
Chronic kidney disease	1 (2.5)
Pulmonary disease	8 (20.0)
Immunological deficits^*c*^	1 (2.5)
Clinical characteristics at ICU_MILANO_ admission	
Previous steroid therapy, no. (%)^*d*^	32 (80.0)
Standard dose	29/32 (90.6)
High dose	3/32 (9.4)
Previous antibiotic exposure, no. (%)^*e*^	30 (75.0)
BL/BLI	15/30 (50.0)
3-4GC	13/30 (43.3)
CARBA	1/30 (3.3)
CST	1/30 (3.3)
TGC	1/30 (3.3)
FQ	5/30 (16.7)
ML	10/30 (33.3)
VAN	5/30 (16.7)
LZD	3/30 (10.0)
Previous MDRO colonization/infection, no. (%)	4 (10.0)
Length of hospital stay before ICU_MILANO_ admittance, days (IQR)	5 (2–11)
PaO_2_/FiO_2_ ratio at ICU_MILANO_ admission, value (IQR)	112 (92–158)
Clinical and microbiological characteristics during ICU_MILANO_ stay	
Timing of CRAB isolation, no. (%)	
≤48 h from ICU_MILANO_ admission	7 (17.5)
>48 h from ICU_MILANO_ admission	33 (82.5)
Pattern of CRAB acquisition of 1st isolate per patient, no. (%)^*f*^	
Infection	14 (35.0)
Colonization	26 (65.0)
For CRAB isolation >48 h from admission, length of ICU_MILANO_ stay before acquisition, days (IQR)	
Infection	12 (6–25.5)
Colonization	11 (6–20)
	15 (6.7–30)
Body site/sample type of CRAB isolation of 1st isolate per patient, no. (%)	
Bronchoalveolar lavage fluid	4 (10.0)
Endotracheal aspirate	19 (47.5)
Surveillance swab (axilla, groin, rectum)	17 (42.5)
Severity of CRAB infection^*f*^	
No sepsis	4/18 (22.2)
Sepsis	7/18 (38.9)
Septic shock	7/18 (38.9)
Antibiotic regimens used for CRAB infections (definitive therapy)^*e*^	
SAM+CST+TGC	5/15 (33.3)
SAM+FDC+CST	1/15 (6.6)
SAM+FDC+CST+TGC	1/15 (6.6)
FDC+CST+TGC	2/15 (13.3)
FDC+TGC	2/15 (13.3)
CST+TGC	4/15 (26.6)
Outcomes	
Death, no. (%)	
During ICU_MILANO_ stay	14 (35.0)
Attributable to CRAB infection during ICU_MILANO_ stay	9/14 (64.3)
During hospitalization	16 (40.0)
Length of ICU_MILANO_ hospitalization, days (IQR)	36.5 (17.5–47.7)
Patients alive at discharge, no. (%)	38 (17.2–54)
Patients dead at discharge, no. (%)	32 (17.5–45.7)
Length of hospitalization, days (IQR)	41 (30–53)
Patients alive at discharge	47 (27.7–61.7)
Patients dead at discharge	39 (30.5–50.2)

a General abbreviations: CRAB, carbapenem-resistant *A. baumannii*; BMI, body mass index; ICU, intensive care unit; MDROs, multidrug-resistant organisms; PAO_2_, partial pressure of oxygen; FIO_2_, fraction of inspired oxygen. Drug abbreviations: BL/BLI, β-lactams/β-lactamase inhibitors; 3-4GC 3rd/4th-generation cephalosporins; CARBA, carbapenems; CST, colistin; TGC, tigecycline; FQ, fluoroquinolones; ML, macrolides; VAN, vancomycin; LZD, linezolid; SAM, ampicillin-sulbactam; FDC, cefiderocol.

b Categorical variables are expressed as frequency (percentages), while continuous variables are expressed as median (interquartile range).

c Category includes at least 1 of the following: solid organ transplantation, active neoplastic disease, hematological disease, rheumatological disease, AIDS, asplenia, chemotherapy in the past 3 months, neutropenia (<500 neutrophils/μL), use of biologics, use of corticosteroids (>10 mg/day prednisone or equivalent >3 months prehospitalization), and other forms of immunosuppression (including congenital/genetic forms).

d The standard dose for use of dexamethasone or methylprednisolone is <1 mg/kg/day, and the high dose for use of methylprednisolone is ≥1 mg/kg of body weight/day or equivalent. (Note that patients could have received both standard and high doses of steroid.)

e Patients could have received more than one class of antibiotic.

f Four patients had the first CRAB isolate as colonization and subsequently developed infection.

Prior to admission to ICU_MILANO_ (the ICU of the Fondazione IRCCS Ca’ Granda Ospedale Maggiore Policlinico di Milano), 32/40 patients (80%) had received steroid therapy, while 30/40 (75%) had received antibiotic therapy—mostly β-lactam/β-lactamase inhibitor (15/30 [50%]), 3rd/4th-generation cephalosporins (13/30 [43%]), and macrolides (10/30 [33%]). Only one patient was exposed to carbapenems. Four patients were diagnosed with MDRO colonization prior to ICU_MILANO_ admission; all had been transferred from the ICU of the same hospital. At entry into ICU_MILANO_, all patients were intubated and mechanically ventilated, with a median partial pressure of oxygen/fraction of inspired oxygen (PAO_2_/FIO_2_) ratio of 112 mm Hg (Q1 to Q3, 92 to 158 mm Hg). The median length of hospital stay before ICU_MILANO_ admission was 5 days (Q1 to Q3, 2 to 11 days).

In 7/40 (17.5%) patients, CRAB was isolated at the first microbiological surveillance (≤48 h from admission) and therefore represented acquisition of infection before the arrival at ICU_MILANO_. The first CRAB isolate represented an infection in 14/40 (35%) patients and a colonization in the remaining 26/40 (65%) patients. Four colonized patients (15.4%) developed CRAB infection during the ICU_MILANO_ stay, with a median time of 14 days (Q1 to Q3, 7 to 18 days). Considering only CRAB isolated >48 h from ICU_MILANO_ admission, the median time between arrival and first pathogen isolation was 12 days (Q1 to Q3, 6 to 25.5 days). All 18 CRAB infections were ventilator-associated pneumonia, with pathogen isolation in the bronchoalveolar lavage fluid specimen and/or endotracheal aspirate. Of them, 4/18 (22.2%) presented without sepsis, 7/18 (38.9%) with signs of sepsis, and 7/18 (38.9%) with septic shock. Only 2/18 (11.1%) were complicated with secondary bacteremia. All but three infected patients underwent antibiotic therapy, with exceptions due to death or transfer to other ICUs before the availability of the microbiological report. All treatments were composed of multidrug regimens, with 4/15 (26.6%) treated with two sequential antibiotic schemes. In accordance with the study period and literature data available that time, the most frequent combination therapies were ampicillin-sulbactam plus colistin plus tigecycline and colistin plus tigecycline (5/15 [33.3%] and 4/15 [26.6%], respectively). Cefiderocol was employed as part of combination therapy in 6/15 (40%) cases.

During ICU_MILANO_ stay, 14/40 (35%) patients died; 9 of them (64.3%) died from sepsis or septic shock attributable to CRAB infection. The overall length of ICU_MILANO_ hospitalization was 36.5 days (Q1 to Q3, 17.5 to 47.7) days, with 38 days (Q1 to Q3, 17.2 to 54 days) for patients alive at discharge and 32 days (Q1 to Q3, 17.5 to 45.7 days) for deceased patients.

### CRAB outbreaks and containment strategies.

**FIG 1:**
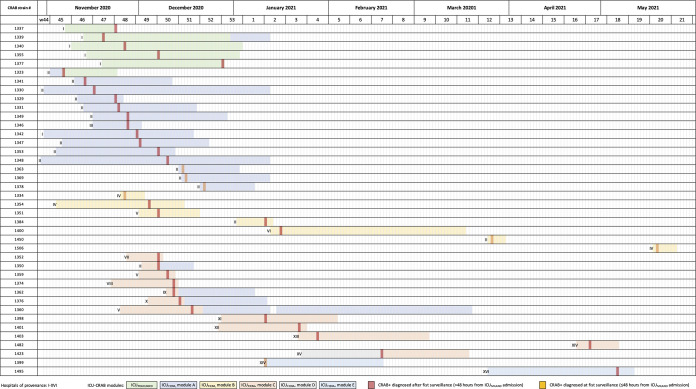
ICU_MILANO_ stay of CRAB patients (*n* = 40) from admission to discharge, including information on hospital of provenance, ICU module, and day of CRAB isolation.

The first recognized case of CRAB was a 56-year-old male patient hospitalized for COVID-19 pneumonia on 27 October 2020 in the Infectious Diseases Unit of a large hospital in the Milan area (CRAB strain 1323) (Fig. 1). Due to severe respiratory failure, on 2 November he was intubated and transferred first to the ICU of the same hospital and later on the same day to ICU_FIERA_ (the Milano Fiera COVID-19 ICU). At that time, extensive CRAB circulation was affecting that referring hospital, and the result of microbiological surveillance performed at hospital ICU admission was positive for CRAB. Unfortunately, due to the emergency situation during the pandemic period, communication of the result to ICU_FIERA_ failed. At ICU_FIERA_, the patient underwent a first routine microbiological surveillance on 2 November, which resulted negative for MDROs. (No specific CRAB surveillance was ongoing at that time.) On 6 November, the patient was centralized to ICU_POLICLINICO_ (the ICU of the Fondazione IRCCS Ca’ Granda Ospedale Maggiore Policlinico di Milano) because of a pneumomediastinum with surgical indication. The result of endotracheal aspirate (ETA) surveillance performed at ICU_FIERA_ the same day of transfer was positive for CRAB, with culture results available 3 days after the sampling. After diagnosis, the patient was promptly put on strict contact precautions, and active screening strategies and IPC measures were implemented in both ICUs. The patient did not develop subsequent CRAB infection and was discharged alive on 23 November 2020.

From November 2020 to May 2021, 39 other patients were found positive for CRAB. After the first recognized case of CRAB isolation in ICU_MILANO_, a specific IPC program was implemented consisting of the following 5 different actions:

1. Active extended surveillance for CRAB. This was done by collecting for all patients swabs from skin (axilla and groin), the pharynx, and rectum at admission and biweekly thereafter. Screening samples were performed using selective MacConkey agar plates (bioMérieux, Florence, Italy) with meropenem (10-μg) disks.
2. Improving behavioral IPC measures. This included staff training sessions on the correct use of personal protective equipment (PPE) and hand hygiene, conducted by trained IPC nurses.
3. Improving environmental IPC measures. This included reinforcement and monitoring of disinfection processes of rooms, areas, and touch surfaces at contact with patients and personnel.
4. Temporary closure of the ICU module and conversion to the CRAB-ICU. After the identification of 2 contemporary cases of CRAB colonization/infection in a single ICU module, that module underwent temporary closure with the suspension of new admissions and conversion to CRAB-ICU. Physical cohorting was applied within the module, with CRAB-infected/colonized patients separated from the other patients, defined as close contacts. In cases of isolation of CRAB within other modules, patients were transferred to the CRAB-ICU.
5. Dynamic ICU module reopening. Reopening of CRAB-ICUs was allowed only after (i) discharge of all CRAB-infected/colonized patients, (ii) negativity of at least 3 consecutive surveillance screenings (performed weekly) of the close contacts, (iii) terminal cleaning of rooms, areas, and surfaces of the ICUs, and (iv) negative environmental swabs for CRAB (including bed rails, infusion pumps, and computer keyboards).

Figure 1 depicts the ICU_MILANO_ stay of CRAB patients from admission to discharge, including information on hospital of provenance, ICU module, and day of CRAB isolation. The majority of CRAB diagnoses occurred from November 2020 to January 2021 (35/40 [87.5%]), while in the 4 following months only 5 cases were found, despite the fact that 45% of all patients were admitted during that period (248/547) (Fig. S2). Six ICU modules were involved in three extensive circulations of CRAB within ICU_MILANO_ (Fig. 1): 5 patients in ICU_POLICLINICO_ and 14, 7, 11, 1, and 2 in modules A, B, C, D, and E of ICU_FIERA_, respectively. Two ICU_FIERA_ modules did not result in any CRAB isolation. In December 2020 and January 2021, environmental surveillance was carried out on surfaces and equipment of ICU_FIERA_ modules A, B, and C (the patient’s bed, echograph, computer keyboard and screen, mechanical ventilator and filters, and ICU trolley). No environmental CRAB colonization was detected.

### Bacterial typing and common characteristics.

Whole-genome assemblies of the isolates displayed a median number of contigs of 120 (Q1 to Q3, 109 to 130), with a median *N*_50_ of 154,380 bp (Q1 to Q3, 131,057 to 161,483 bp), while the isolates’ total genome sizes ranged from 3.86 to 4.07 Mb. By multilocus sequence typing (MLST), all sequenced isolates belonged to the Pasteur sequence type 2 (ST2), while they belonged to five different STs by the Oxford scheme, namely, ST451/1809 (17/40 [42.5%]), ST218/2164 (10/40 [25.0%]), ST208/1806 (7/40 [17.5%]), ST369 (4/40 [10.0%]), and ST425 (2/40 [5.0%]). Typing of the capsular polysaccharide (K) and lipooligosaccharide outer core loci (OCL) revealed the presence of 5 different K locus (KL) variants, clustering in accordance with Oxford STs—i.e., KL12 (17/40 [42.5%]), KL7 (10/40 [25.0%]), KL2 (7/40 [17.5%]), KL9 (4/40 [10.0%]), and KL40 (2/40 [5.0%])—and only one OCL variant, OCL-1.

All strains shared a set of antimicrobial resistance and virulence genes, including the acquired carbapenemase gene *bla*_OXA-23_, always found on a Tn*2006* transposon flanked by two copies of an IS*Aba1* insertion sequence, together with genes conferring resistance to aminoglycosides [*aph(3′′)-Ib* and *aph(6)-Id*], fluoroquinolones (*abeM* and *mexT*), macrolides (*abeS*), and tetracyclines (*tetA*), multidrug efflux pumps of the resistance-nodulation-division (RND) family (*adeABC* and *adeJKL*), as well as core virulence factors, usually displayed by Acinetobacter baumannii strains (19–21) (see Data Set S1 in the supplemental material). The antimicrobial resistance genes observed were concordant with the MIC values obtained for the strains (Table S1).

### A. baumannii relatedness according to maximum likelihood, minimum spanning trees, and whole-genome signatures.

**FIG 2:**
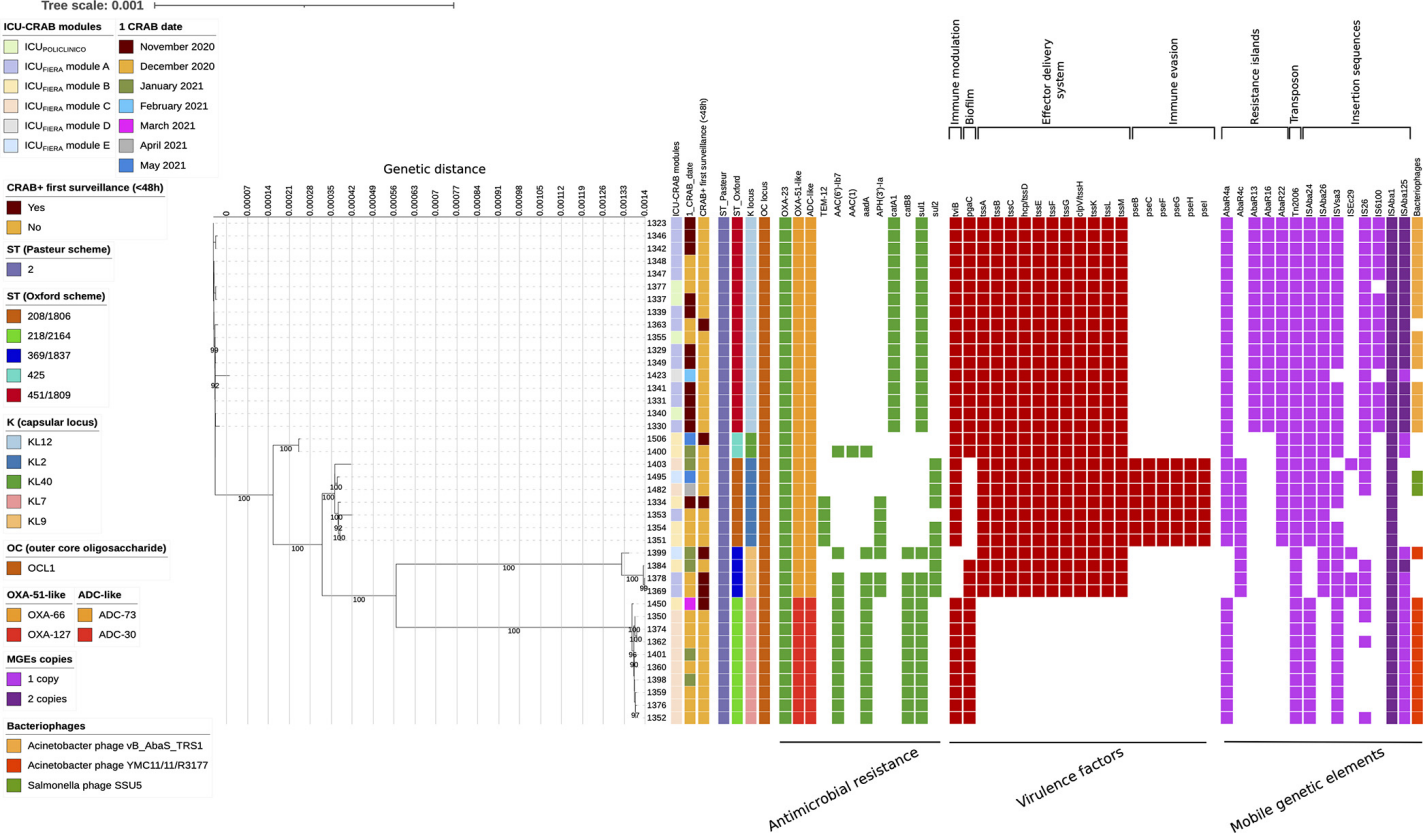
Estimated maximum likelihood phylogenetic analysis of Acinetobacter baumannii isolates (*n* = 40) in an ICU COVID-19 hub hospital in Italy. The maximum likelihood was inferred from a core genome alignment of 3,078,653 bp. The phylogeny was estimated with IqTree using the best-fit model of nucleotide substitution TIM2+F+R3 with 1,000 replicates and fast bootstrapping. The numbers on leaves represent the sample IDs, and bootstrap values higher than 90 are shown on branches. Information regarding the samples were reported: the ICU module (ICU-CRAB module), date of isolation (1 CRAB date), if the positivity appeared at the first surveillance, within 48 h from the entry in the ICU (CRAB^+^ first surveillance), the sequence type (ST), capsular locus (K locus) and lipooligosaccharide outer core (OC locus), and the presence (solid squares) or absence of antimicrobial resistance and virulence genes and mobile genetic elements (MGEs). Core antimicrobial resistance and virulence genes, shared by all strains, are not reported in the figure.

The maximum likelihood (ML) phylogenetic tree, inferred from a core genome alignment of 3,078,653 bp, confirmed the clustering based on the Oxford STs and on the K capsular locus distribution (Fig. 2), revealing the presence of 5 clusters with a bootstrap value of >90%.

In particular, the biggest cluster comprised 17 strains belonging to ST451/1809, characterized by a median intracluster single nucleotide polymorphism (SNP) distance of 3 (Q1 to Q3, 1 to 7) (Fig. S3A and B). Of these 17 sequences, 15 presented a number of intracluster SNPs of <10 (median [Q1 to Q3], 2 [1 to 3]), while 2 sequences (IDs 1363 and 1423) presented a number of intracluster SNPs of ≥10 (median [Q1 to Q3], 22 [11 to 23]) (Fig. S3A and B, in red). When looking at the SNP localization of these 2 strains, we found that they were all scattered throughout the core genome, rather than being concentrated in a single region, excluding the acquisition of core genome portions through recombination events as the reason for the increased core SNP distance. Besides the core resistome and virulome characterizing all the A. baumannii strains as previously described, whole-genome analysis revealed that all ST451/1809 strains shared further antimicrobial resistance (AMR) and virulence genes, including the OXA-66 (OXA-51-like variant) and ADC-73 genes, as well as mobile genetic elements (Fig. 2). The main differences detected were in strain 1363, which did not carry the vB AbaS_TRS1 phage, and strain 1423 which did not carry IS*Vsa3*, IS*6100*, and the vB AbaS_TRS1 phage and possessed only one copy of IS*Aba125*. Of note, these results were concordant with the genetic divergence of strains 1363 and 1423 detected by core SNP analysis. IS*6100* was not carried also by strain 1377, characterized by an intracluster distance of median 6 (IQ1 to IQ3, 5 to 7).

The smallest cluster identified comprised the 2 strains (IDs 1400 and 1506) belonging to ST425. These strains differed from each other by 18 core SNPs and presented similar AMR and virulence genes, as well as mobile genetic elements (MGEs), with the exception of genes *aac(6′)-Ib7*, *aac(1)*, and *aadA*, conferring resistance to aminoglycosides, which were present only in strain 1400 (Fig. 2).

The cluster comprising the 7 strains belonging to ST208/1806 was characterized by a median intracluster SNP distance of 27 (Q1 to Q3, 20 to 68) (Fig. S3C and D). Among the 7 sequences, 3 presented a number of intracluster SNPs lower than 10 (median [Q1 to Q3], 7 [5 to 8]), while 4 sequences (IDs 1353, 1403, 1482, and 1495) presented a number of intracluster SNPs of ≥10 (median [Q1 to Q3], 39 [23 to 69]) (Fig. S3C and D, in red), all found to be interspersed along the core genome. Whole-genome analysis was concordant with core SNP data, as it revealed that the 4 strains displaying a higher number of intracluster SNPs differed in genetic content, like AMR genes and MGEs, compared to the other strains. In particular, strain 1353 did not carry the *sul2* gene, conferring resistance to sulfonamides, and the MGE IS*Vsa3*. Strain 1403 carried the MGEs IS*Ec29* and IS*26*, while it lacked IS*Vsa3*. Strains 1482 and 1495 carried the MGEs IS*26* and Salmonella phage SSU5. Of note, the genes coding for the β-lactamase TEM-12 and APH(3′)-Ia, conferring resistance to aminoglycosides, were shared by the 3 strains with a number of core SNPs lower than 10 (IDs 1334, 1354, and 1351), and by strain 1353, characterized by an intracluster core SNP distance of ≥10.

The fourth cluster identified comprised 4 strains belonging to ST369/1837 and was characterized by a median intracluster SNP distance of 55 (Q1 to Q3, 7 to 102) (Fig. S3E and F). Among the 4 sequences, 3 presented a number of intracluster SNPs lower than 10 (median [Q1 to Q3], 5 [7 to 8]), while only one sequence (ID 1399) presented a number of intracluster SNPs of ≥10 (median [Q1 to Q3], 102 [102 to 103]) (Fig. S3E and F, in red). Also in this case, SNPs were not concentrated in a single region, but rather dispersed throughout the core genome. Whole-genome analysis confirmed the low genetic relatedness between strain 1399 and the other 3 strains, due to the loss of the *pgaC* biofilm gene, and the insertion sequence IS*26*. Whole-genome analysis also revealed that all strains shared the same AMR and virulence genes, except for strain 1384. This strain did not carry genes *aac(6′)-Ib7*, *aph(3′)-Ia*, and *aadA*, conferring resistance to aminoglycosides, the *catB8* gene and *sul1*, conferring resistance to phenicols and sulfonamides, respectively, and insertion sequence IS*Ec29*. Of note, these genes and ISs were all carried by a single plasmid (homologous to plasmid p2BJAB07104 [accession no. CP003907.1]), suggesting the loss of this mobile genetic element in strain 1384 and thus indirectly confirming its close genetic relatedness to strains 1369 and 1378.

Finally, the last cluster identified comprised 10 strains belonging to ST218/2164 and was characterized by a median intracluster SNP distance of 3 (Q1 to Q3, 2 to 6) (Fig. S3G and H). Among the 10 sequences, 9 presented a number of intracluster SNPs lower than 10 (median [Q1 to Q3], 3 [2 to 4]), while only one sequence (ID 1450) presented a number of intracluster SNPs of ≥10 (median, [Q1 to Q3], 34 [34 to 34] (Fig. S3G and H, in red), with all SNPs found to be distributed along the core genome. Whole-genome analysis revealed that all strains shared genes coding for OXA-127 and ADC-30, known to be OXA51-like and ADC-like variants. These variants were exclusively found in these ST218/2164 strains. The only difference identified (besides the SNPs in the core genome) was IS*26* carried by strains 1350, 1362, 1352, and 1450.

### Characterization of transmission chains.

**FIG 3:**
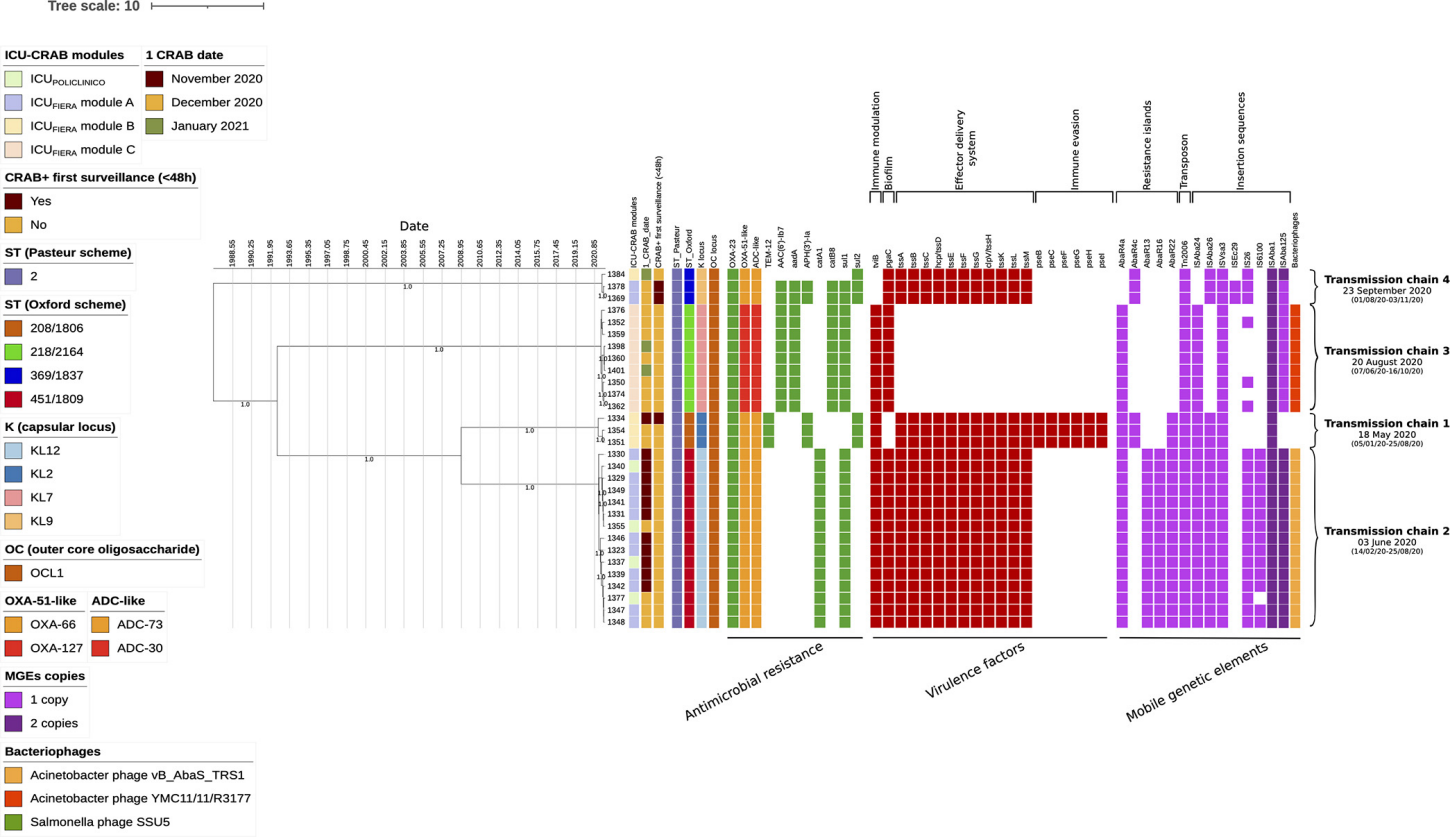
Bayesian reconstruction incorporating the date of first positivity of the 30 Acinetobacter baumannii isolates grouped in transmission chain clusters. The Bayesian method was inferred from a core genome alignment of 3,078,653 bp. The Bayesian phylogeny was estimated with BEAST by running 3 independent chains for 10 million states, using the best-fit model of nucleotide substitution GTR+G4 with a strict molecular clock and an exponential population growth tree prior. The numbers on leaves represent the sample IDs, posterior probabilities of >0.9 are shown on branches. Information regarding the samples were reported: the ICU module (ICU-CRAB module), date of isolation (1 CRAB date), if the positivity appeared at the first surveillance, within 48 h from the entry in the ICU (CRAB^+^ first surveillance), the sequence type (ST), capsular locus (K locus), and lipooligosaccharide outer core (OC locus), and the presence (solid squares) or absence of antimicrobial resistance and virulence genes and mobile genetic elements (MGEs). Core antimicrobial resistance and virulence genes, shared by all strains, are not showed in the figure. Transmission chains are reported numbered from the oldest to the most recent based on the putative age root (with 95% HPD) calculated with Tracer.

From the results obtained by the ML tree, minimum spanning tree, SNP distance, and whole-genomic analysis, it was evident the presence of 4 putative transmission chains, constituted by ≥3 strains with an intracluster pairwise SNP distance always lower than 10 (22–24). Hence, a Bayesian analysis was performed to better characterize these putative transmission chains, after the removal of a total of 10 sequences (IDs 1363, 1423, 1403, 1495, 1482, 1353, 1399, 1400, 1450, and 1506), differing for a number of core SNPs of ≥10 compared to the strains of the same ML cluster. Bayesian phylogenetic analysis was performed after a good correlation was found by looking at the root-to-tip versus sampling time regression of the core genome of the 30 remaining isolates, confirming the strength of the temporal signal (Fig. S4). The Bayesian phylogenetic analysis also resulted in effective sample size (ESS) values always higher than 200 (Table S2), confirming the existence of four potential transmission routes, composed of 3, 9, 3, and 15 strains, respectively, circulating between November and January 2021 (Fig. 3).

The 3 strains belonging to ST369/1837 (transmission chain 4) (Fig. 3) were found to be part of the most recent putative transmission chain, with the origin traced back to 23 September 2020 (95% highest posterior density [HPD] of 1 August 2020 to 3 November 2020). Of these, two strains (1378 and 1369) were isolated in ICU_FIERA_ module A on 16 and 22 December 2020 at the first surveillance screening (<48 h from admission), suggesting acquisition outside ICU_FIERA_. Strain 1384, on the other hand, was isolated from ICU_FIERA_ module B on 11 January 2021, more than 48 h from admission. Strain 1384 was characterized by 7 core SNPs, the most informative localized in genes involved in lipopolysaccharide (LPS) biosynthesis (KpsF/GutQ isomerase; A1881713T with respect to the core genome), SOS mutagenesis (*recA*; T2487885A with respect to the core genome) and lipid A modification (i.e., the two-component system sensor histidine kinase PmrB; T2447800A and T2447971G with respect to the core genome). By whole-genome analysis, we found that this strain lost the plasmid homologous to plasmid p2BJAB07104, resulting in the loss of *aac(6′)-Ib7*, *aph(3′)-Ia*, *aadA*, *catB8*, *sul1*, and IS*Ec29*, as previously described. These modifications could have occurred during 2 months of ST369/1837 persistence in the two ICU_FIERA_ modules, even though the acquisition of 1384 by a different transmission route cannot be epidemiologically excluded.

The 9 strains belonging to ST218/2164 (transmission chain 3) (Fig. 3) were all isolated between December 2020 and January 2021 from ICU_FIERA_ module C more than 48 h from admission, suggesting the acquisition inside the ICU_FIERA_. The origin of this cluster was traced back to 20 August 2020 (95% HPD, 6 June 2020 to 16 October 2020), while the putative index isolates, giving rise to the transmission chain, were hypothesized to be either strain 1350 or 1352, since first positivity was detected for both strains on 7 December 2020. Nonetheless, tracing the exact chain of transmission of this cluster is hampered by the absence of a clear strain of origin, also leaving open the possibility of the acquisition of CRAB from an unknown source outside ICU_FIERA_. All the strains shared the same AMR and virulence genes, as well as MGEs, emphasizing their genetic relatedness. This was also confirmed by the low number of core SNPs among the strains (always lower than 6).

The 3 strains belonging to ST208/1806 (transmission chain 1) (Fig. 3) were part of a putative transmission chain most likely originating from strain 1334, which was detected in the ICU_FIERA_ module B at the first surveillance screening (<48 h from admission) on 26 November 2020 and thus probably acquired outside ICU_FIERA_. The putative acquisition outside ICU_FIERA_ might also be supported by the older age root of this cluster compared to the others (8 May 2020 with 95% HPD of 5 January 2020 to 25 August 2020). The isolation of strain 1334 was followed by the detection of strains 1354 and 1351, which were isolated inside the same ICU on 4 and 7 December 2020, respectively, after 48 h from admission. These strains shared all AMR and virulence genes, as well as MGEs, and differed for only a few SNPs, most of which fell in a plasmid replication protein (A694029T, G694080T, C694231T, and A694270C with respect to the core genome).

The last 15 strains, belonging to ST451/1809 (transmission chain 2) (Fig. 3), were isolated between November and December 2020 inside ICU_FIERA_ module A and ICU_POLICLINICO._ One of the strains of this cluster, strain 1323, represented the first recognized case of CRAB in ICU_MILANO_. In fact, this strain entered ICU_FIERA_ on the 2 November 2020 and was then transferred to ICU_POLICLINICO_ on 6 November, putatively causing the spread of CRAB in other 14 individuals across both ICUs. In line with the isolation of strain 1323 in early November 2020, the origin of this transmission chain was traced back to 3 June 2020 (14 February 2020 to 25 August 2020). All sequences shared the same AMR and virulence genes, as well as MGEs, with the exception of strain 1377, which did not carry the insertion sequence IS*6100*. Strain 1377 was also characterized by 5 intracluster SNPs (Fig. S3B), randomly localized in the core genome. Of note, the isolation of strain 1377 occurred in late December (isolation date of 28 December 2020), 18 days later than the second-to-last isolate (ID 1348, isolation date of 10 December 2020). Even if the genomic insights on 1377 and its localization in ML and Bayesian trees can suggest its involvement in the transmission chain 2, its acquisition by a different route cannot be epidemiologically excluded.

No transmission chains were detected after January 2021, despite the fact that 45% of all patients were admitted from February to May 2021 (Fig. S2).

## DISCUSSION

This study reports a wide circulation of carbapenemase-resistant Acinetobacter baumannii in a large COVID ICU hub of Milan during the second wave of the COVID-19 pandemic, which was successfully contained by the IPC measures implemented. Moreover, the refined genomic characterization of circulating A. baumannii strains and the investigation of potential relatedness and transmission chains through whole-genome sequencing (WGS) and core genome alignment allowed us to better characterize their circulation dynamics.

In line with what was reported in the literature (25, 26), our cohort was characterized by intense levels of support and severe clinical condition, indicated by both the severity of respiratory failure at ICU_MILANO_ admission and the high mortality rates. Despite the short hospital stay before ICU_MILANO_ admittance, with a median of only 5 days of hospitalization, the majority of patients were exposed to steroids and broad-spectrum antibiotics (80% and 75%, respectively), which are known risk factors for CRAB acquisition (25, 27). Over the last years, several IPC measures have been recommended to manage CRAB outbreaks in the ICU. A recent review of 12 studies conducted over the last 10 years in settings where CRAB transmission is both epidemic and endemic found wide variability in the frequency of application of IPC measures (28). While all studies adopted a multimodal approach, the most frequently applied strategies (75 to 100% of studies) resulted in environmental disinfection, contact precautions, and cohorting of staff and patients. Conversely, daily chlorhexidine baths, active rectal screening, and ICU closure were adopted in less than 60%, one-half and just one-third of the studies, respectively. Interestingly, WGS analysis was employed only in 25% of the studies (29). In their study conducted before COVID-19, Meschiari et al. proposed a multimodal approach that yielded promising results without cohorting, admission restriction, or ICU closure (18). A central step in their bundle was the “cycling environmental cleaning and disinfection” procedure, which is described as very effective yet labor-intensive and therefore difficult to apply in a high-risk and low-resource scenarios.

The CRAB outbreak described in our report occurred in a critical phase of COVID-19 pandemic in Italy, when the high pressure on hospitals, particularly ICUs, posed IPC strategies at significant risk of failure. Factors such low staffing, scarce adherence to PPE, inadequate environmental cleaning, wide antibiotic use, and prolonged critical illness of mechanically ventilated COVID-19 patients may all have contributed to increase the likelihood of MDRO transmission (28).

In this regard, the whole-genome characterization performed on CRAB isolates revealed that all strains belonged to the same MLST Pasteur sequence type (ST2), which is the most disseminated clone globally, while they belonged to five different Oxford STs (ST451/1809, ST218/2164, ST208/1806, ST369, and ST425). All strains shared the *bla*_OXA-23_ gene (21), always found on a Tn*2006* element, flanked by two copies of the IS*Aba1* insertion sequence. In addition, all strains presented genes conferring resistance to other antimicrobial agents, like aminoglycosides, fluoroquinolones, macrolides, and tetracyclines, and core virulence factors (19, 20). The ML analysis confirmed the clustering of the strains based on the five Oxford STs and allowed identification of differences in AMR, virulence, and MGEs among and within the different STs. Based on the ML and minimum spanning trees obtained by the core genome and through the definition of a threshold of 10 core SNPs (a threshold concordant with the literature in references 22 to 24), we were able to identify four putative transmission chains, involving over two-thirds of CRAB strains (30/40). The genetic relatedness of these strains was also confirmed by the fact that they almost always shared the same AMR and virulence genes and mobile genetic elements.

A more in-depth characterization of these putative transmission chains was then obtained by including these 30 strains in a Bayesian analysis, taking into account the date of first positivity. This analysis confirmed the existence of four transmission chains circulating between November 2020 and January 2021 inside and among the ICUs. By integrating these genomic analyses with the patients’ data, the following epidemiological scenario could be hypothesized. Strain 1323, which represented the first recognized case of CRAB in ICU_MILANO_ (isolation date of 2 November 2020) because it was isolated from a patient who entered ICU_FIERA_ module A already positive, putatively caused the spread of CRAB in other 14 individuals across both ICU_POLICLINICO_ and module A of ICU_FIERA_ (transmission chain 2). This strain has not been reported as being detected before 48 h from admission because no specific CRAB surveillance was ongoing at that time inside ICU_FIERA._ However, on the same day of ICU_FIERA_ entry, the patient result was positive for CRAB at the hospital where he was initially admitted. Therefore, a lack of communication of the results between the two hospitals, due to the ongoing COVID-19 emergency, putatively resulted in CRAB transmission inside both ICU_FIERA_ module A and ICU_POLICLINICO_.

The three other transmission chains involved ICU_FIERA_ only (with only one involving two modules of the same ICU) and were less numerous because they were likely contained by IPC measures rapidly introduced after the first evidence of CRAB circulation. Transmission chain 1 was characterized by an origin date quite superimposable on transmission chain 2. It most likely started in ICU_FIERA_ from strain 1334, detected on 26 November 2020 at the first surveillance screening, and thus was acquired most probably outside ICU_FIERA_ and putatively caused the spread of CRAB in other two individuals inside module B of ICU_FIERA_ on December 2020. Transmission chain 3 also consisted of 9 CRAB isolates diagnosed in only one module (module C of ICU_FIERA_) between December 2020 and January 2021. All CRABs were isolated more than 48 h from admission in patients coming from different hospitals, suggesting CRAB acquisition inside module C of ICU_FIERA_. Finally, the most recent transmission chain identified consisted of two strains isolated on December 2020 in ICU_FIERA_ module A less than 48 h from admission, suggesting CRAB acquisition outside ICU_FIERA_, and one strain (ID 1384) isolated from ICU_FIERA_ module B on 11 January 2021, 2 months after the first and more than 48 h from admission, hypothesizing a putative persistence of this CRAB strain in the two modules during the whole period.

It is noteworthy that our work reinforced the idea that an integration of methodologies investigating both the core genome, through ML, MST, and SNP analysis, and the whole genome, by identification of AMR and virulence determinants, as well as mobile genetic elements, is essential to correctly identify and characterize potential transmission chains (22–24). Indeed, the core genome SNP approach alone could fail to detect essential information like loss or acquisition of entire genes, while whole-genome sequencing could be difficult to apply to maximum likelihood or Bayesian methods, especially when a huge number of isolates needs to be studied. To define potential transmission chains, we decided to apply an SNP threshold of <10 among isolates. This threshold is in line with recent published papers (24, 30), even if a little above the 2.5 SNPs proposed by Coll et al. (23). Regarding this point, we need to highlight that the transmission chains here described suffered the introduction of CRAB isolates originating in different hospitals of the Lombardy region and had quite different durations. It is worth notice that the core genome SNP thresholds (<10) considered in our study to infer potential transmission chains among CRAB strains were always concordant with the results obtained by whole-genome inspection. The only 2 sequences for which the core SNP and the whole genome returned slightly different results are IDs 1377 and 1384 (the last detected isolates of transmission chains 2 and 4, respectively), both characterized by an isolation date that occurred 18 days and 2 months later than the second-to-last strain part of the same transmission chain. Moreover, the careful inspection of the whole genome provided evidence that the main differences, with respect to the other sequences of the transmission chains, were a single insertion sequence (IS*6100*), lost in 1377, and an entire plasmid, lost in 1384.

The complete absence of CRAB transmission chains after January 2021 suggests the potential efficacy of the rapidly introduced IPC measures. Our five-model approach consisted of three universal IPC measures (enhanced CRAB screening, environmental disinfection, and reinforcement of behavioral measures, such as hand hygiene and correct PPE use), along with the dynamic closure/conversion to CRAB-ICUs and reopening of ICU modules, a two-step strategy specifically tailored to the modular organization of ICU_MILANO_. Indeed, by applying these measures, we were able to contribute to the reduction on CRAB transmission within the ICU and at the same time maintain the ICU admission rates and occupancy at sufficient levels during a period of high need, as confirmed by trends of admissions per month illustrated in Fig. S2. Unfortunately, we did not have available data on CRAB incidence in each hospital referring patients to ICU_FIERA_ (more than 45 hospitals across the Lombardy region in Northern Italy), so we cannot rule out the possibility that at least part of the reduction in CRAB events may be attributed to a general reduction of CRAB incidence in the area from January 2021 onward.

In conclusion, through the integration of genome analysis with clinical and epidemiological patient data, we documented the potential contribution of a tailored IPC strategy in containing the spread of CRAB within a large modular ICU. Our results underline the potentiality of integrating classical epidemiological studies with genomic investigation to identify the transmission routes during outbreaks, which in turn could be of great value to ensure IPC strategies for preventing the diffusion of MDROs inside the ICU.

## MATERIALS AND METHODS

### Study setting.

The present study includes patients admitted from October 2020 to May 2021 to the ICUs of Fondazione IRCCS Ca’ Granda Ospedale Maggiore Policlinico di Milano (ICU_MILANO_). Since March 2020, to face the impact of COVID-19, the ICU already existing at Fondazione IRCCS Ca’ Granda Ospedale Maggiore Policlinico di Milano (ICU_POLICLINICO_ [27 beds]) was converted to COVID-ICU and an adjunctive ICU was built, ICU_FIERA_ (Milano Fiera COVID-19 ICU). ICU_FIERA_ was a large ICU composed of 7 distinct modules to accommodate up to 100 patients with severe actute respiratory syndrome coronavirus 2 (SARS-CoV-2) infection requiring mechanical ventilation. ICU_FIERA_ modules were open space areas without physical barriers between patients. Each module could accommodate up to 16 beds, with a nurse-to-patient ratio of 1:2. For routine care, personal protective equipment (PPE) of physicians and nurses consisted of FFP2/N95 masks, hazmat suits, face shields/visors, and double gloves. From October 2020 to May 2021, ICU_FIERA_ hosted over 450 mechanically ventilated COVID-19 patients coming from several hospitals in the Milan area and from different health care settings (emergency rooms, nonintensive hospital wards, other ICUs). Despite each module being managed by a different staff, microbiological surveillance was standardized, and all modules referred to the Fondazione IRCCS Ca’ Granda Ospedale Maggiore Policlinico di Milano for microbiological analyses and consultations by infectious disease physicians and hospital epidemiologists, the same way as ICU_POLICLINICO_. Therefore, the term ICU_MILANO_ refers to patients admitted to either one of the two ICUs.

Routine microbiological surveillance was performed in all COVID-19 mechanically ventilated patients accordingly to procedures described in the supplemental material.

In evaluating CRAB isolates, infections were defined by the presence of a significant bacterial load (≥10^5^ CFU/mL on endotracheal aspirate or ≥10^4^ CFU/mL on bronchoalveolar lavage specimens) associated with clinical manifestation within the infection window period (IWP [±3 days from specimen collection]) (31), whereas isolates were classified as indicating colonization when no adverse clinical signs or symptoms were documented.

### Antimicrobial susceptibility testing.

Antimicrobial susceptibility testing was performed by broth microdilution using the Microscan WalkAway (Beckman Coulter, Inc.) for a total of 8 antibiotics, namely, amikacin, ciprofloxacin, gentamicin, imipenem, levofloxacin, meropenem, trimethoprim-sulfamethoxazole, and tigecycline. Susceptible, intermediate, and resistant categories were assigned according to the EUCAST breakpoint table (v.13.0; available at https://www.eucast.org/clinical_breakpoints).

### Definition of CRAB genetic relatedness.

CRAB genetic relatedness was evaluated by a combined approach using whole-genome sequence data and core alignment following the steps described below.

### Bacterial typing and whole-genome analysis.

Whole-genome sequencing (WGS) was performed and data assembled as described in the supplemental material. In order to proceed with the bacterial typing, multilocus sequence typing (MLST) was performed with the mlst tool (v.2.11) (32, 33), using the Pasteur and Oxford scheme (34, 35). The capsular polysaccharide loci (KL) and outer core lipooligosaccharide loci (OCL) were assessed by Kaptive (v.0.7.3) (36). Investigation of antibiotic resistance (AMR) genes was carried out with ABRicate (v.0.4), by using the Comprehensive Antibiotic Resistance Database (CARD) (37) and ResFinder database (38), while virulence factors were investigated using the Virulence Factor Database (VFDB) (39). The MobileElementFinder tool (v.1.0.3) (40), was used for identification of mobile genetic elements (MGEs), while the presence of intact bacteriophages was investigated using PHASTER (41, 42). The identification of AbaR-type genomic islands (AbaRs) was performed by searching in the assembled contigs for 59 AbaRs (43) using BLAST. Plasmids were inferred from contigs using the MOB suite tool (44, 45). All these analyses were performed on the whole genome.

### Core genome and phylogenetic analysis.

To explore the concordance between ST distribution and CRAB clustering and to identify potential transmission chains, an approach based on core genome alignment was used. Core genome analysis was performed with Roary (v.3.13.0) (46), with default parameters, obtaining a core genome alignment shared by 95% of the isolates. This core genome alignment was then inspected by maximum likelihood (ML) and minimum spanning tree (MST) methods as described in the supplemental material. In accordance with the genetic relatedness of the 40 A. baumannii strains, a threshold of 10 SNPs was considered suggestive of potential transmission chains. This threshold is consistent with already published articles on bacterial divergence, which consider a minimum of 2.5 and maximum of 15 core genome SNPs to rule out transmission chains (22–24).

Furthermore, to better characterize CRAB transmission chains, the core genomes of CRAB strains (i) clustering together in a number ≥3 in the ML phylogenetic tree with a bootstrap value higher than 90% and (ii) characterized by a pairwise intracluster SNP distance of <10 compared to the other strains in the same ML cluster were incorporated in a Bayesian tree inference together with information about the date of first positivity. The Bayesian coalescent tree analysis was undertaken with BEAST (v.1.10.4) (30, 47–50) as detailed in the supplemental material.

### Ethical and regulatory aspects.

Clinical and epidemiological data from the study population in analysis were retrieved from two COVID-19 studies conducted at Fondazione IRCCS Ca’ Granda Ospedale Maggiore Policlinico of Milano and already approved by the Hospital Advisory Board (Comitato Etico Milano Area 2; protocols 0008489 and 0025505-U) and preregistered at clinicaltrials.gov (identifiers NCT04388670 and NCT05293418). Written informed consent was waived because of the retrospective nature of the analysis.

### Data availability.

The 40 Acinetobacter baumannii sequence data obtained in this study are openly available on European Nucleotide Archive (ENA) under accession no. ERR10500712 to ERR10500715, ERR10500872 to ERR10500883, ERR10500972 to ERR10500988, ERR10500991, and ERR10500993 to ERR10500998. A database including selected clinical and epidemiological data and genome analysis of CRAB strains of the study population is available as Data Set S1 in the supplemental material. The Beast.xml file used to infer the Bayesian tree is available as Data Set S2, deposited in Zenodo (https://zenodo.org/record/7645944#.Y-3-2tLMKo5).
