# Supplementary material for: Genomic Characterization of Carbapenem-Resistant Acinetobacter baumannii (CRAB) in Mechanically Ventilated COVID-19 Patients and Impact of Infection Control Measures on Reducing CRAB Circulation during the Second Wave of the SARS-CoV-2 Pandemic in Milan, Italy
Source: Microbiol Spectr. 2023 Mar 28;11(2):e00209-23. doi: 10.1128/spectrum.00209-23 (PMC10100775; doi:10.1128/spectrum.00209-23)
Supplement: Supplemental file 2 — Supplemental material. Download spectrum.00209-23-s0002.pdf, PDF file, 2.0 MB [file spectrum.00209-23-s0002.pdf]

Supplementary Materials

Routine microbiological surveillance

Routine microbiological surveillance consisted in active screening of MDROs colonization through: i) nasal swabs (selective culture media for methicillin-resistant Staphylococcus aureus - MRSA) and rectal swabs (selective culture media for vancomycin-resistant Enterococcus faecium and Enterococcus faecalis – VRE and for extended spectrum betalactamases-producing (ESBLs) and carbapanemases-producing Enterobacterales) performed at admission and on weekly basis, ii) endotracheal aspirates cultures (EACs) performed at admission and on bi-weekly basis. CRAB-specific screening consisted in the addition of skin surface (axilla and groin), pharynx and rectal swabs (Mac-Conkey agar and CHROMID® CARBA agar for Acinetobacter baumannii MDR).

Whole genome sequencing (WGS)

Genomic DNA was extracted from *A. baumannii* pure isolates using ZymoBIOMICS DNA Miniprep Kit (Zymo Reseach) in accordance with manufacturer's instructions. Libraries for whole genome sequencing were generated using Illumina DNA Library Prep kit (Illumina, Inc., San Diego, CA, USA). PCR products, approximately sized 600 base-pairs, were visualized with Bioanalyzer 2100 and High Sensitivity DNA kit (Agilent, Santa Clara, CA, USA). Finally, libraries were sequenced on Illumina MiSeq sequencing platform (Illumina, San Diego, CA, USA) using MiSeq Reagent Kit v2.

Reads pre-processing, assembly and annotation

Raw reads were trimmed for adapters and filtered for quality (average phred score>20) with Fastp (v0.20.1) (1) and quality checked after trimming with FastQC (v0.11.9) (2). A *de novo* whole genome assembly was performed using SPAdes (v3.14.1) (3), with the '-careful' option, and quality of the assemblies was evaluated by Quast (v5.1) (4). Annotation of the assembled contigs was performed with Prokka (v1.14.6) (5)

Phylogenetic analysis

Maximum likelihood and Minimum Spanning Trees

Maximum likelihood (ML) tree was constructed on the core genome alignment by IQTREE (v2.0.6) (6) with 1000 bootstrap replicates under the best nucleotide substitution model TIM2+F+R3, inferred by ModelFinder (7). Minimum Spanning trees were constructed on the core genome alignment with Grapetree (v1.5.0) (8) and pairwise SNP distances were calculated using snp-dists tool (https://github.com/tseemann/snp-dists).

Bayesian phylogenetic analysis

In order to better characterize CRAB transmission chains initially identified by ML and MST analyses, the core genome alignment of the informative strains was incorporated in a Bayesian tree inference obtained by BEAST (v1.10.4) (9), using the information about the date of first positivity and the best substitution model GTR+G4, inferred by bModelTest (10), after checking for the regression of dates of sampling against the root-to-tip distances with TempEst (v1.5.3) (11). Three independent BEAST runs were performed using a strict molecular clock, prior set as 'exponential population growth', and Monte Carlo Markov Chain (MCMC) iteration set as 10 millions. BEAST outputs were then combined and Tracer (v1.7.1) (12) was used to evaluate that the Effective Sample Sizes (ESS) were always higher than 200. Posterior trees were combined with LogCombiner and summarized in TreeAnnotator after a 10% burn-in. Phylogenetic trees were visualized and annotated using iTOL (v5) (13).

References Supplementary Materials

1. Chen S, Zhou Y, Chen Y, Gu J. fastp: an ultra-fast all-in-one FASTQ preprocessor. Bioinformatics. 2018;34(17):i884-i890
2. Andrews S. FastQC: a quality control tool for high throughput sequence data. 2010. Available online at: http://www.bioinformatics.babraham.ac.uk/projects/fastqc

3. Bankevich A, Nurk S, Antipov D, et al. SPAdes: a new genome assembly algorithm and its applications to single-cell sequencing. J Comput Biol. 2012;19(5):455-477
4. Gurevich A, Saveliev V, Vyahhi N, Tesler G. QUAST: quality assessment tool for genome assemblies. Bioinformatics. 2013;29(8):1072-1075
5. Seemann T. Prokka: rapid prokaryotic genome annotation. Bioinformatics. 2014;30(14):2068-2069
6. Nguyen LT, Schmidt HA, von Haeseler A, Minh BQ. IQ-TREE: a fast and effective stochastic algorithm for estimating maximum-likelihood phylogenies. Mol Biol Evol. 2015;32(1):268-274
7. Kalyaanamoorthy S, Minh BQ, Wong TKF, von Haeseler A, Jermiin LS. ModelFinder: fast model selection for accurate phylogenetic estimates. Nat Methods. 2017;14(6):587-589
8. Zhou Z, Alikhan NF, Sergeant MJ, et al. GrapeTree: visualization of core genomic relationships among 100,000 bacterial pathogens. Genome Res. 2018;28(9):1395-1404
9. Suchard MA, Lemey P, Baele G, Ayres DL, Drummond AJ, Rambaut A. Bayesian phylogenetic and phylodynamic data integration using BEAST 1.10. Virus Evol. 2018;4(1):vey016
10. Bouckaert RR, Drummond AJ. bModelTest: Bayesian phylogenetic site model averaging and model comparison. BMC Evol Biol. 2017;17(1):42
11. Rambaut A, Lam TT, Max Carvalho L, Pybus OG. Exploring the temporal structure of heterochronous sequences using TempEst (formerly Path-O-Gen). Virus Evol. 2016;2(1):vew007
12. Rambaut A, Drummond AJ, Xie D, Baele G, Suchard MA. Posterior Summarization in Bayesian Phylogenetics Using Tracer 1.7. Syst Biol. 2018;67(5):901-904
13. Letunic I, Bork P. Interactive Tree Of Life (iTOL) v5: an online tool for phylogenetic tree display and annotation. Nucleic Acids Res. 2021;49(W1):W293-W296

SUPPLEMENTARY INFORMATION LEGENDS

Supplementary Table 1. Minimal inhibitory concentration (MIC) values of the tested antibiotics.

Supplementary Table 2. Effective Sample Sizes (ESS) values of the Bayesian analysis outputted by Tracer.

Supplementary Figure 1. Flow chart of the study population for those CRAB strains were isolated and characterized for genomic content by Maximum Likelihood tree and Bayesian phylogenetic analysis.

Supplementary Figure 2. Admission trend by month in ICU$_{FIERA}$ (Panel A) and ICU$_{POLICLINICO}$ (Panel B) and proportion of admitted patients who developed at least one CRAB event during their ICU stay.

Supplementary Figure 3. Pairwise core SNPs distances of *A. Baumannii* strains against Maximum Likelihood clusters. Analyses were inferred from a core genome alignment of 3,078,653 bp. The left side of the Figure reports the scatter plots showing the intra- and inter-cluster distances for the Maximum Likelihood (ML) clusters ST451/1809 (A), ST208/1806 (C), ST369/1837 (E) and ST218/2164 (G) with median (IQR) SNP distance reported below. The threshold of 10 SNPs used to infer transmission chains within the ML cluster is reported with a dotted line. P-values were calculated with the Mann-Whitney test. The right side of the Figure reports the Minimum Spanning Trees of the pairwise distance within the ML clusters ST451/1809 (B), ST208/1806 (D), ST369/1837 (F) and ST218/2164 (H). Strains with a SNP distance lower than 10 (green) were considered as part of transmission chains and included in the Bayesian phylogenetic analysis.

Supplementary Figure 4. Root-to-Tip Genetic Distance for CRAB core sequences characterized by an intra-cluster distance <10 SNPs plotted against collection date. The graph was inferred from a core genome alignment of 3,078,653 bp. The Pearson correlation coefficient between root-to-tip distance and collection date is 0.618.

Supplementary Data 1. Database including selected clinical-epidemiological data and genome analysis of CRAB strains of the study population.

Supplementary Table 1. Minimal inhibitory concentration (MIC) values of the tested antibiotics

Sample	AMK	MIC AMK	CIP	MIC CIP	GEN	MIC GEN	IPM	MIC IPM	LVX	MIC LVX	MEM	MIC MEM	SXT	MIC SXT	MIC TGC
1376	R	>16	R	>1	R	>4	R	>8	R	>1	R	16	R	>4/76	<=1
1348	R	>16	R	>1	R	>4	R	>8	R	>1	R	16	R	>4/76	<=1
1330	R	>16	R	>1	R	>4	R	>8	R	>1	R	>32	R	>4/76	2
1342	R	>16	R	>1	R	>4	R	>8	R	>1	R	>32	R	>4/76	>2
1323	R	>16	R	>1	R	>4	R	>8	R	>1	R	>32	R	>4/76	>2
1377	R	>16	R	>1	R	>4	R	>8	R	>1	R	>32	R	>4/76	>2
1340	R	>16	R	>1	R	>4	R	>8	R	>1	R	>32	R	>4/76	>2
1354	R	>16	R	>1	R	>4	R	>8	R	>1	R	>32	R	>4/76	>2
1353	R	>16	R	>1	R	>4	R	>8	R	>1	R	32	R	>4/76	>2
1337	R	>16	R	>1	R	>4	R	>8	R	>1	R	>32	R	>4/76	>2
1347	R	>16	R	>1	R	>4	R	>8	R	>1	R	>32	R	>4/76	<=1
1341	R	>16	R	>1	R	>4	R	>8	R	>1	R	>32	R	>4/76	<=1
1329	R	>16	R	>1	R	>4	R	>8	R	>1	R	32	R	>4/76	<=1
1331	R	>16	R	>1	R	>4	R	>8	R	>1	R	>32	R	>4/76	<=1
1339	R	>16	R	>1	R	>4	R	>8	R	>1	R	>32	R	>4/76	<=1
1355	R	>16	R	>1	R	>4	R	>8	R	>1	R	>32	R	>4/76	<=1
1349	R	>16	R	>1	R	>4	R	>8	R	>1	R	>32	R	>4/76	<=1
1346	R	>16	R	>1	R	>4	R	>8	R	>1	R	>32	R	>4/76	<=1
1374	R	>16	R	>1	R	>4	R	>8	R	>1	R	>32	R	>4/76	1
1334	R	>16	R	>1	R	>4	R	>8	R	>1	R	32	R	>4/76	<=1
1360	R	>16	R	>1	R	>4	R	>8	R	>1	R	32	R	>4/76	2

1352	R	>16	R	>1	R	>4	R	>8	R	>1	R	32	R	>4/76	1
1351	R	>16	R	>1	R	>4	R	>8	R	>1	R	16	R	>4/76	<=1
1359	R	>16	R	>1	R	>4	R	>8	R	>1	R	>32	R	>4/76	<=1
1350	R	>16	R	>1	R	>4	R	>8	R	>1	R	16	R	>4/76	<=1
1362	R	>16	R	>1	R	>4	R	>8	R	>1	R	32	R	>4/76	<=1
1363	R	>16	R	>1	R	>4	R	>8	R	>1	R	>32	R	>4/76	<=1
1369	R	>16	R	>1	R	>4	R	>8	R	>1	R	32	R	>4/76	<=1
1378	R	>16	R	>1	R	>4	R	>8	R	>1	R	32	R	>4/76	<=1
1401	R	>16	R	>1	R	>4	R	>8	R	>1	R	16	R	>4/76	<=1
1398	R	>16	R	>1	R	>4	R	>8	R	>1	R	16	R	>4/76	2
1384	S	<=8	R	>1	S	<=2	R	>8	R	>1	R	32	R	>4/76	<=1
1399	R	>16	R	>1	R	>4	R	>8	R	>1	R	32	R	>4/76	1
1400	R	>16	R	>1	R	>4	R	>8	R	>1	R	32	S	<=2/38	<=1
1403	R	>16	R	>1	R	>4	R	>8	R	>1	R	32	R	>4/76	<=1
1423	R	>16	R	>1	R	>4	R	>8	R	>1	R	>32	R	>4/76	<=1
1495	R	>16	R	>1	R	>4	R	>8	R	>1	R	>32	R	>4/76	<=1
1450	R	>16	R	>1	R	>4	R	>8	R	>1	R	16	R	>4/76	1
1482	R	>16	R	>1	R	>4	R	>8	R	>1	R	>32	R	>4/76	2
1506	R	>16	R	>1	R	>4	R	>8	R	>1	R	16	I	>4/76	<=1

Abbreviations: AMK=amikacin, CIP=ciprofloxacin, GEN=gentamicin, IPM=imipenem, LVX=levofloxacin, MEM=meropenem, SXT=trimethoprim-sulfamethoxazole, TGC=tigecycline, S=susceptible, I=intermediated, R=resistance. Susceptible, intermediate and resistant categories were assigned according to the EUCAST breakpoint table (version 13.0, available at https://www.eucast.org/clinical_breakpoints).

Supplementary Table 2. Effective Sample Sizes (ESS) values of the Bayesian analysis outputted by Tracer.

Statistic	Mean	95% HPD interval	ESS	Type
joint	-4267000	-4266093.9103, -4266057.8073	14859	R
prior	-93.185	-113.7453, -75.3506	336	R
likelihood	-4267000	-4265991.0416, -4265972.2236	14830	R
treeModel.rootHeight	36.362	20.1088, 58.2792	405	R
age(root)	1984.696	1962.7783, 2000.9487	405	R
treeLength	120.955	67.9247, 195.0034	405	R
tmrca(cluster1)	0.677	0.4062, 1.0444	577	R
tmrca(cluster2)	0.633	0.4062, 0.9351	561	R
tmrca(cluster3)	0.421	0.2653, 0.6233	609	R
tmrca(cluster4)	0.328	0.2157, 0.4727	828	R
age(cluster1)	2020.38	2020.0132, 2020.6513	577	R
age(cluster2)	2020.425	2020.1224, 2020.6513	561	R
age(cluster3)	2020.636	2020.4342, 2020.7922	609	R
age(cluster4)	2020.729	2020.5848, 2020.8418	828	R
exponential.popSize	2.876	1.0824, 5.2913	740	R
exponential.growthRate	-0.162	-0.2842, -0.046	1085	R
gtr.rates.rateAC	0.46	0.4151, 0.4974	327	R
gtr.rates.rateAG	1.752	1.6906, 1.8249	386	R
gtr.rates.rateAT	0.451	0.4169, 0.4831	427	R
gtr.rates.rateCG	0.361	0.3136, 0.3999	285	R
gtr.rates.rateCT	2.542	2.4745, 2.6265	290	R
gtr.rates.rateGT	0.433	0.3932, 0.4694	349	R
frequencies1	0.299	0.2989, 0.2999	6355	R
frequencies2	0.188	0.1876, 0.1885	5300	R
frequencies3	0.214	0.2139, 0.2148	6246	R
frequencies4	0.298	0.2977, 0.2987	4850	R
alpha	0.02512	2.7993E-3, 0.0488	10399	R
clock.rate	0.0001218	9.9397E-6, 2.8601E-5	15024	R
meanRate	0.0001218	9.9397E-6, 2.8601E-5	15024	R
treeLikelihood	-4267000	-4265991.0416, -4265972.2236	14830	R
branchRates	0	n/a	-	*
coalescent	-70.503	-88.0074, -55.2687	337	R

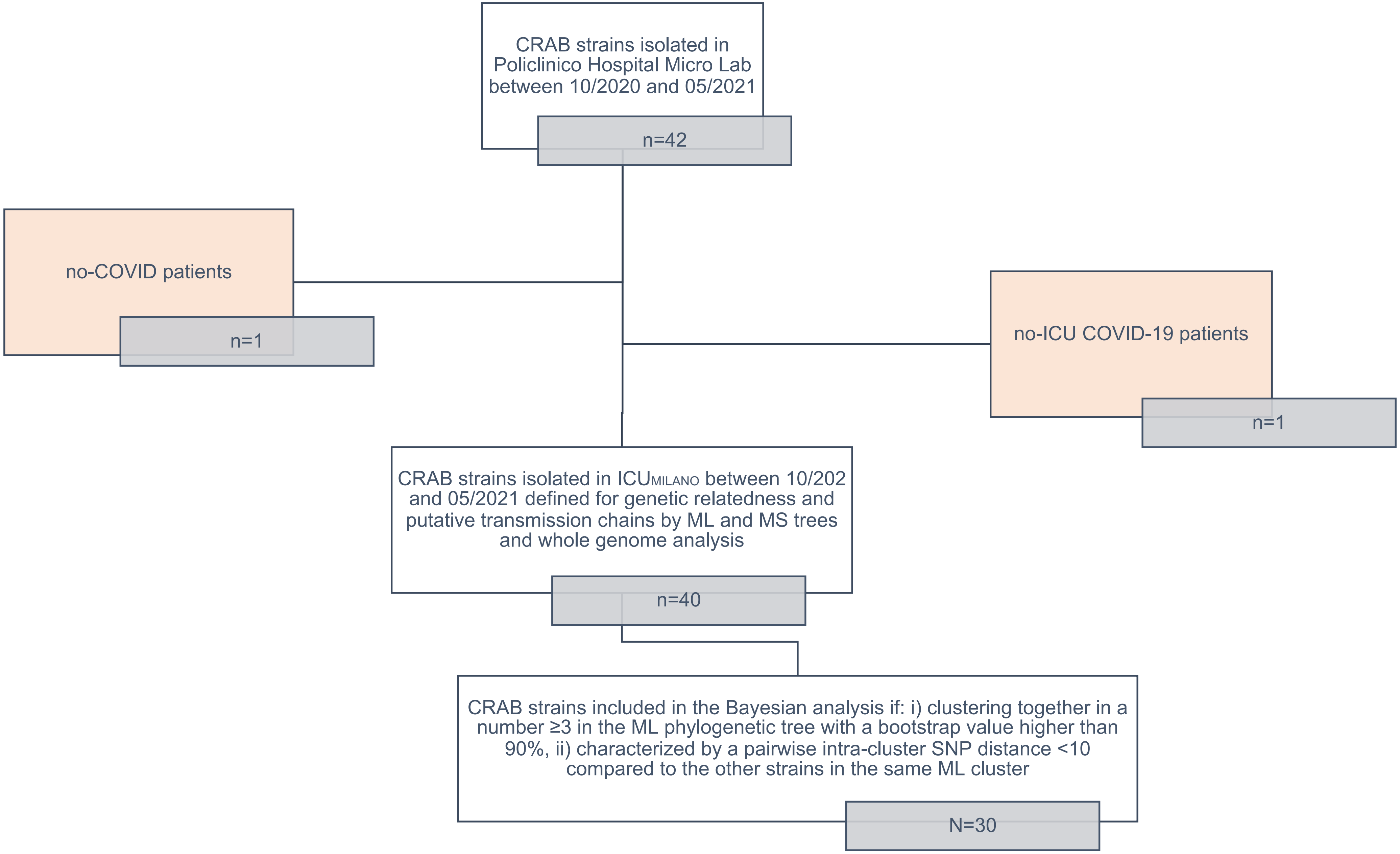

Supplementary Figure 1. Flow chart of the study population for those CRAB strains were isolated and characterized for genomic content by Maximum Likelihood tree and Bayesian phylogenetic analysis.

A

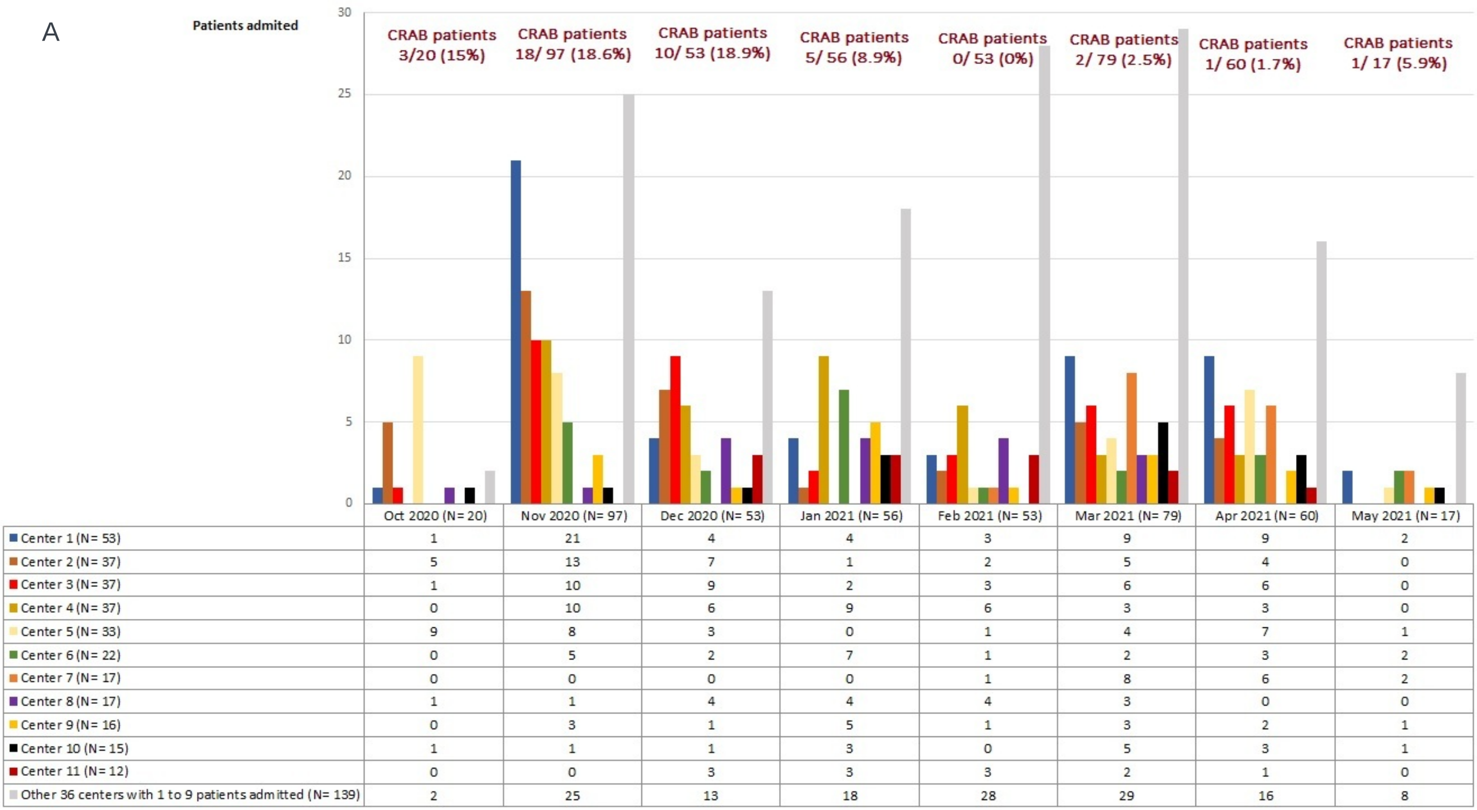

	Oct 2020 (N= 20)	Nov 2020 (N= 97)	Dec 2020 (N= 53)	Jan 2021 (N= 56)	Feb 2021 (N= 53)	Mar 2021 (N= 79)	Apr 2021 (N= 60)	May 2021 (N= 17)
Center 1 (N= 53)	1	21	4	4	3	9	9	2
Center 2 (N= 37)	5	13	7	1	2	5	4	0
Center 3 (N= 37)	1	10	9	2	3	6	6	0
Center 4 (N= 37)	0	10	6	9	6	3	3	0
Center 5 (N= 33)	9	8	3	0	1	4	7	1
Center 6 (N= 22)	0	5	2	7	1	2	3	2
Center 7 (N= 17)	0	0	0	0	1	8	6	2
Center 8 (N= 17)	1	1	4	4	4	3	0	0
Center 9 (N= 16)	0	3	1	5	1	3	2	1
Center 10 (N= 15)	1	1	1	3	0	5	3	1
Center 11 (N= 12)	0	0	3	3	3	2	1	0
Other 36 centers with 1 to 9 patients admitted (N= 139)	2	25	13	18	28	29	16	8

B

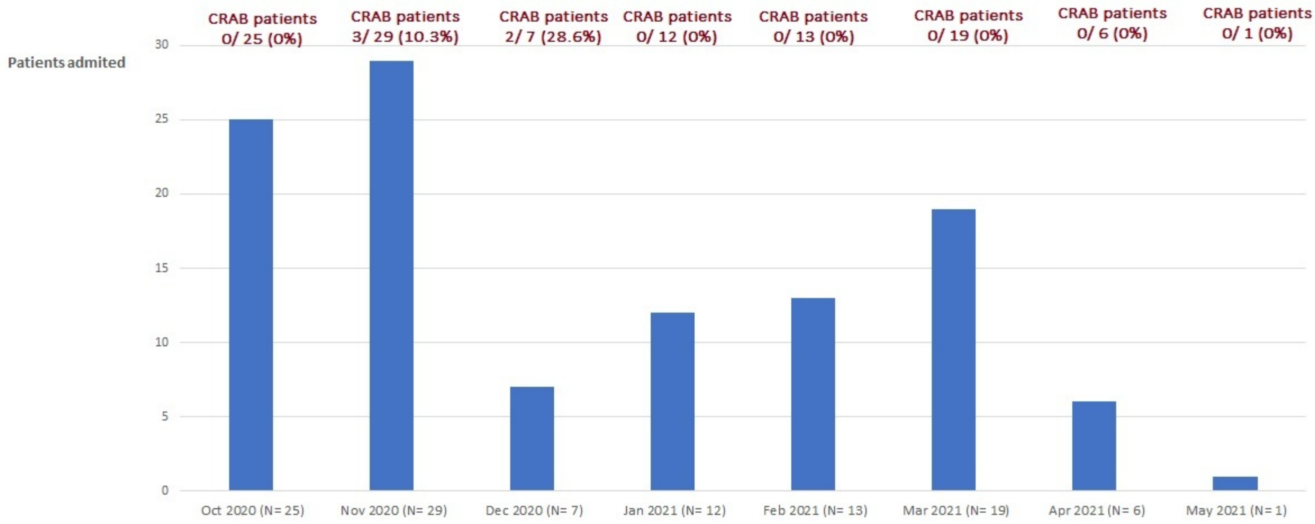

Supplementary Figure 2. Admission trend by month in ICUFIERA (Panel A) and ICUPOLICLINICO (Panel B) and proportion of admitted patients who developed at least one CRAB event during their ICU stay.

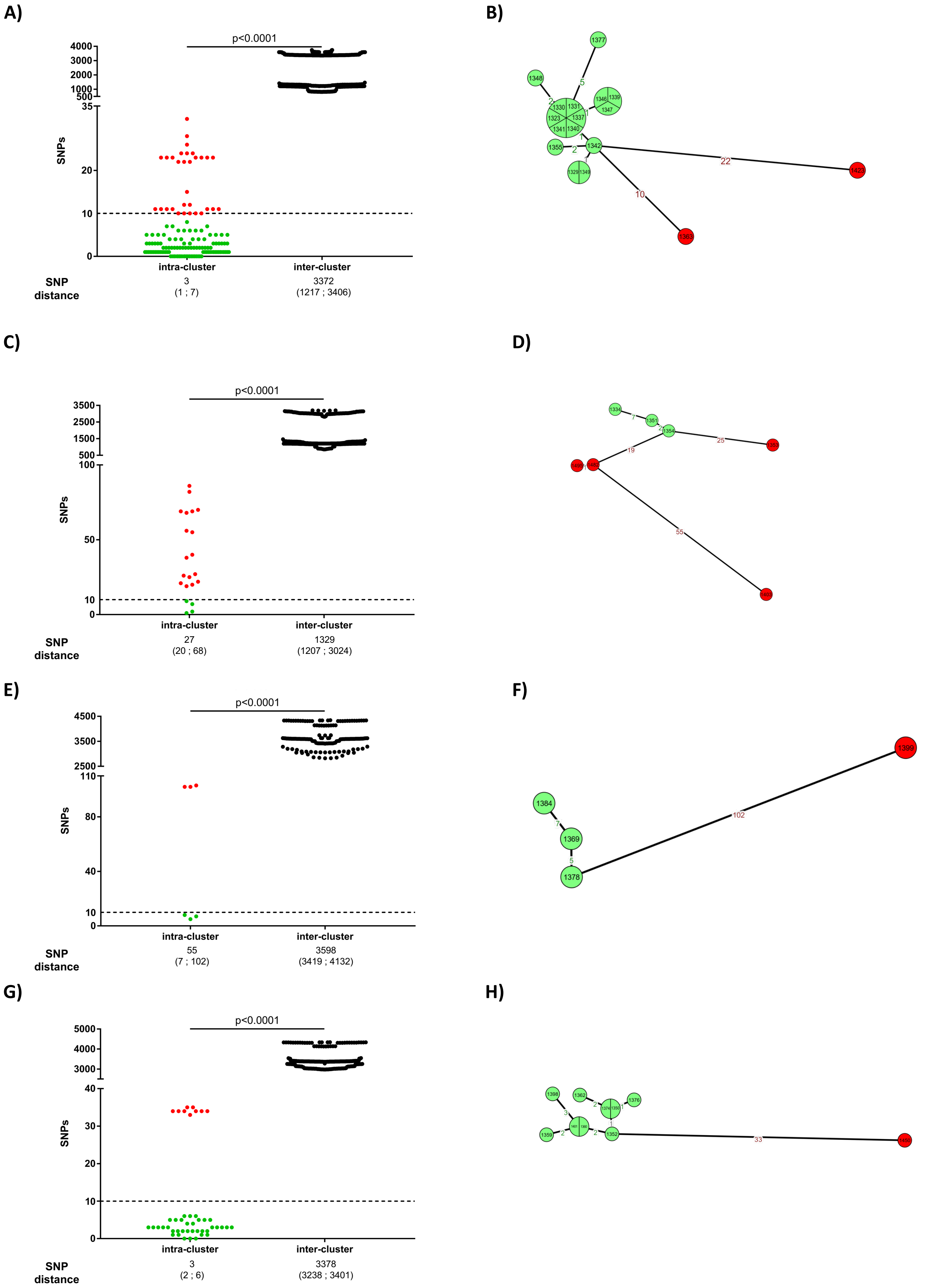

Supplementary Figure 3. Pairwise core SNPs distances of A.Baumannii strains against Maximum Likelihood clusters. Analyses were inferred from a core genome alignment of 3,078,653 bp. The left side of the Figure reports the scatter plots showing the intra- and inter-cluster distances for the Maximum Likelihood (ML) clusters ST451/1809 (A), ST208/1806 (C), ST369/1837 (E) and ST218/2164 (G) with median (IQR) SNP distance reported below. The threshold of 10 SNPs used to infer transmission chains within the ML cluster is reported with a dotted line. P-values were calculated with the Mann-Whitney test. The right side of the Figure reports the Minimum Spanning Trees of the pairwise distance within the ML clusters ST451/1809 (B), ST208/1806 (D), ST369/1837 (F) and ST218/2164 (H). Strains with a SNP distance lower than 10 (green) were considered as part of transmission chains and included in the Bayesian phylogenetic analysis.

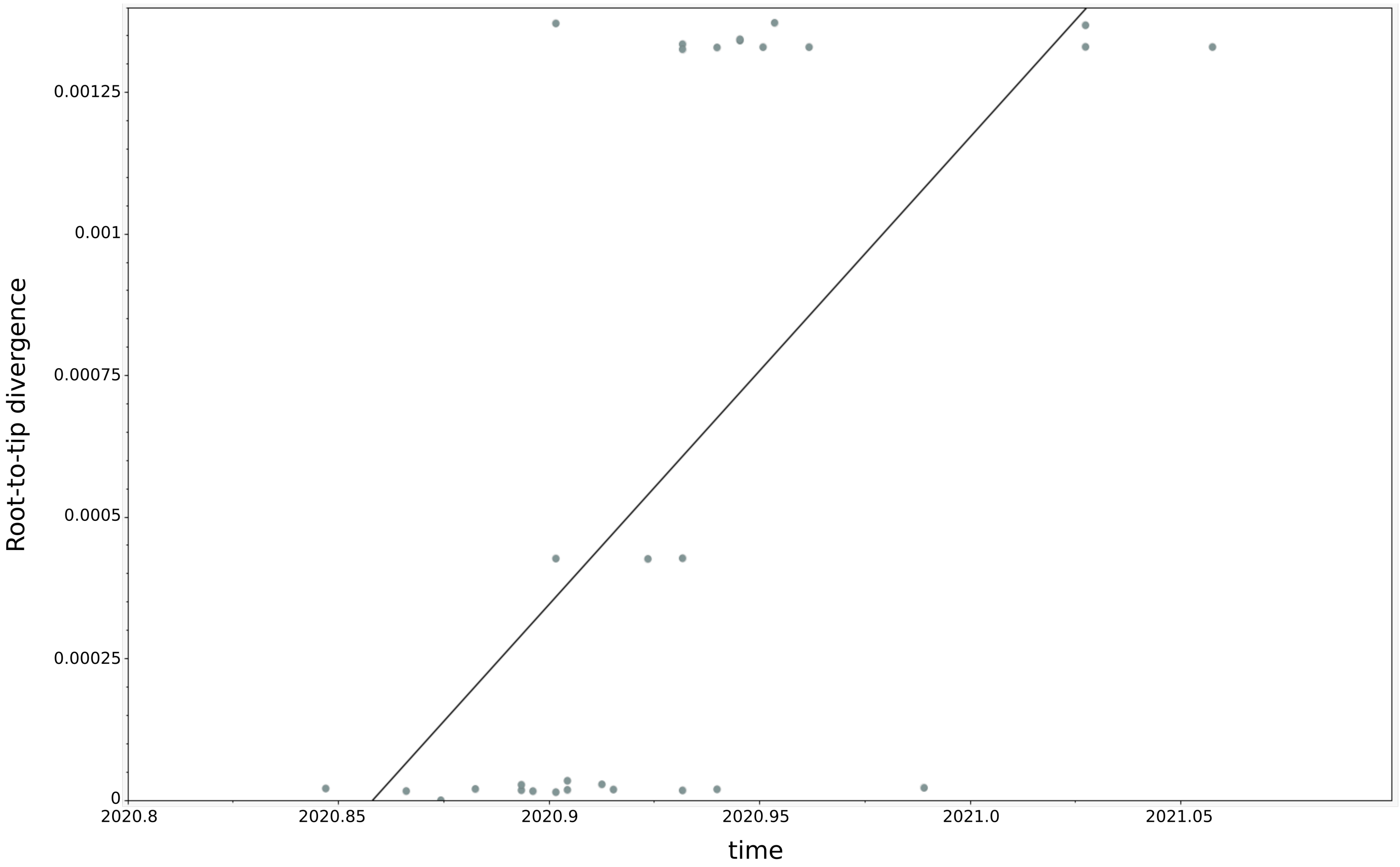

Supplementary Figure 4. Root-to-Tip Genetic Distance for CRAB core sequences characterized by an intra-cluster distance <10 SNPs plotted against collection date. The graph was inferred from a core genome alignment of 3,078,653 bp. The Pearson correlation coefficient between root-to-tip distance and collection date is 0.618.
